# The Utility of Early Warning Score in Adults Presenting With Sepsis in the Emergency Department of a Low Resource Setting

**DOI:** 10.7759/cureus.9030

**Published:** 2020-07-06

**Authors:** Emaduddin Siddiqui, Abdul A Jokhio, Ahmed Raheem, Shahan Waheed, Syed Hashmatullah

**Affiliations:** 1 Emergency Medicine, Aga Khan University Hospital, Karachi, PAK; 2 Emergency Medicine, Dow University of Health Sciences, Karachi, PAK; 3 Pathology and Laboratory Medicine, Aga Khan University Hospital, Karachi, PAK; 4 Emergency Department, Aga Khan University Hospital, Karachi, PAK; 5 Psychiatrist Adult & Addiction Services, Grey Bruce Health Services, Ontario, CAN

**Keywords:** sepsis, mortality, morbidity, septic shock, prognosis, severe sepsis

## Abstract

Background

Sepsis is a condition with high mortality and morbidity. Delay in early recognition and prompt management results in higher mortality. There are many clinical scores to identify early sepsis; however, Early Warning Score (EWS) has clinical/physiological parameters that are easy to apply in the ED for timely diagnosis and management. In the present study, we collected information regarding the utilization of EWS in timely identifying the sick patients at triage of a tertiary care center.

Methods

This study was a descriptive cross-sectional investigation conducted in the ED of Aga Khan University Hospital in Karachi, the largest metropolitan city in Pakistan. A total of 240 participants were selected by non-probability convenient sampling after fulfilling the inclusion criteria. Data collected included EWS criteria, demography, length of hospital stay, patient disposition (ward, intensive care or high dependency area), and differentials like sepsis, severe sepsis or septic shock.

Results

A total of 240 patients were enrolled, out of which 139 (57.9%) patients were male, and 101 (42.1%) were female with a mean age of 52.7 ± 15.3 years (range: 18 to 80 years). In this study, the length of stay (LOS) was 2.2 ± 1.1 (range: one to six days), and there was an EWS of 8.2 ± 2.6 (4-15). There were 143 patients in the elderly age group > 50 years (59.6%); however, most elderly presented with sepsis among both age groups. The least affected age group was aged 16 to 30 years, with 23 (9.6%) cases. An EWS >7 is best to detect cases with sepsis or severe sepsis with a sensitivity of 98.5% (95% CI: 92.13 to 99.92) and specificity of 89.57% (95% CI: 82.64 to 93.93). Similarly, the EWS for severe sepsis or septic shock was >9 with a sensitivity of 86.76% (95% CI: 76.72 to 92.88) and specificity of 88.24% (95% CI: 78.47 to 93.92).

Conclusions

This study revealed that the sensitivity and specificity of EWS for the detection of sepsis, severe sepsis and septic shock was found to be high; hence, it could be a valuable and readily useable system for early diagnosis and proper management of sepsis, severe sepsis, and septic shock.

## Introduction

Observational studies suggest that the clinical signs of deterioration may present as early as 24 hours before presentation to the hospital as a serious clinical event [1]. Sepsis is a condition with high mortality and morbidity [2,3]. Local sepsis data from urban settings demonstrate a mortality rate of 23% [4]. Sepsis tends to progress very rapidly, and a delay in early recognition and treatment can result in higher mortality [5]. More rapid administration of antibiotics also reduces overall mortality from septic shock, as recommended by the Surviving Sepsis Campaign [4]. However, since the introduction of early goal-directed therapy, mortality significantly reduces to 7% to 16% in certain hospitals [6].

There are multiple criteria for sepsis identification in clinical use, with most using both clinical and laboratory parameters that may not be useful in early identification [7-14]. Early Warning Score (EWS) is a tool based on clinical and physiological criteria [15]. Hence, it is easy to use and track sepsis within the busy ED. The score has shown promise in early detection and management of septic patients, resulting in good outcomes.

EWS, based on previous similar scoring systems, is comprised of seven physiological parameters, each of which is assigned a value between 0 and 3 along with an additional parameter for supplemental oxygen, which scores zero or two [12-14]. The score for each of these seven parameters is summed to calculate the EWS, which may range between 0 and 15: the higher the score, the greater the deviation from normality [15].

EWS is a good modality to identify early sepsis at triage as it contains basic parameters which may be measured easily in a short time even in a remote setting with just a basic vital monitor and a junior nurse. Similar scores like national early warning score (NEWS) or NEWS-2 include Carbon dioxide (Co2) which is difficult to measure at triage. In our setting, emergency severity index (ESI) v 4.0 is used as a standard of care and all basic parameters already embedded in our system. ESI is a good tool to identify sick patient; however, ESI III is the most grey area where a larger number of patients fall who may either be more sick or not at all, hence another handy, easy to use system is the need to differential and identify septic patient at triage with more precision.

In this study, we aim to assess whether EWS may identify sepsis, severe sepsis, and septic shock very early at ED presentation, which may help in the early identification of sick patients with timely management of their condition, hence reducing morbidity and mortality.

## Materials and methods

This was a single-center cross-sectional study, conducted at the ED of Aga Khan University Hospital in Karachi, Pakistan. The duration of the study was approximately six months between May 11, 2016 and November 10, 2016.

The participants were selected through a nonprobability consecutive sampling technique. The prevalence of sepsis in the intensive care units of Pakistan is reported to be 7% [4]. Using the World Health Organization sample size calculator, with a 95% CI, and 3% margin of error, the estimated sample size was calculated to be 240.

Patients aged 18 years and older of either sex attending the critical area of ED with a temperature of either > 38.3°C or < 36.0°C, a pulse > 90 beats per minute, a respiratory rate > 20 breaths per minute, and an oxygen saturation of < 94% along with presumed or confirmed infection (confirmed cases as per the culture results of patient body fluids or other signs of sepsis-like pneumonia, meningitis or obvious cellulitis etc, while presumed sepsis are all those cases with fever but no obvious focus of infection) were included in our study. Once the participants fulfilled the inclusion criteria, a detailed medical history was collected. Severe sepsis and septic shock were categorized as per the Surviving Sepsis Guidelines. Exclusion criteria included pregnancy, poly-trauma, the patient had major surgery in the past 30 days, the patient had prior do-not-resuscitate orders or had a known chronically deliberated disease like chronic kidney disease, a cerebrovascular accident, a seizure disorder, malignancy or the patient was categorized as P3 or P4 as per Emergency Triage Severity (ESI) acuity [16]. Data were collected on a predesigned datasheet after taking written informed consent. EWS consists of physiological parameters such as respiratory rate, temperature, pulse, oxygen saturation, blood pressure, and consciousness level; each of them is given a score for the assessment of seventy for the critical or non-critical patient. The score for each of the seven parameters is summed to calculate the EWS, which may range between zero and 15: the higher the score, the greater the deviation from normality (Table 1). No study-related therapeutic or diagnostic intervention was carried out. In addition, this study had gone through approval by the institutional ethical review with the 42201-EM-ERC-16 board.

**Table 1:** Early warning score *On the AVPU scale: A, alert; V, verbal; P, pain; U, unresponsive [15]

	Early warning score point						
	3	2	1	0	1	2	3
Respiratory rate	<8	-	9-11	12-20	-	21-24	≥25
Oxygen saturation	≤94	92-93	94-95	≥96	-	-	-
Supplemental oxygen	-	Yes	-	No	-	-	-
Pulse rate	≤40	-	41-50	51-90	91-110	111-130	≥130
Systolic blood pressure	≤90	91-100	101-110	111-219	-	-	≥220
Temperature	≤35°	-	35.1-36°	36.1-38°	38.1-39°	≥39°	-
Consciousness level*	-	-	-	A	-	-	V, P, U

Data were analyzed on IBM SPSS Statistics for Windows, Version 21.0. (IBM Corp., Armonk, NY) and Prism GraphPad version 8.1.2 (GraphPad Software, San Diego, CA). Descriptive statistics were reported and included mean along with standard deviation for the age of the patient, the duration of concerns, and early warning score. Proportions and percentages for categorical variables were calculated for gender, sepsis, severe sepsis, and septic shock. The receiver operating characteristic (ROC) curve consists of the ratios obtained from the scores of each cut-off value (threshold), and the ratio that accurately predicts the actual default. True-positive rate and false-positive rate incorrectly predict actual normality as default is represented by graphs corresponding to Y-axis and X-axis coordinates, respectively.

Analysis of the ROC area under the curve (AUC) was calculated to see overall performances for sepsis, severe sepsis, and septic shock with EWS with 95% CI. Optimal cut-off values were chosen to maximize the sum of sensitivity (Se) and specificity (Sp). Positive predictive values, negative predictive values, positive likelihood ratios, and negative likelihood ratios were also assessed. All analyses were performed at 95% CI with a 5% level of significance.

## Results

The mean and standard deviation of age 52.7 ± 15.3 (18 to 80 years), hospital length of stay (LOS) 2.2 ± 1.1 (one to six days), and EWS of 8.2 ± 2.6 (4 to 15), respectively. A higher mean significance value of age was observed in septic shock as compared to severe sepsis (53 ± 14 vs. 59 ± 14, P < .001). Patients who were in severe sepsis had a longer hospital stay as compared to septic shock (2.7 ± 0.8 vs. 2.4 ± 1.5, P < .001). In contrast, we also showed that the value of EWS increased in septic shock (8.8 ± 0.911 vs. 8 ± 1.3, P < .001)

We analyzed the demographic descriptions of the 240 patients enrolled, of whom 139 (57.9%) patients were men, and 101 (42.1%) were women. There were 143 patients in the elderly age group of > 50 years (59.6%). The least affected age group was aged 18 to 30 years, with 23 (9.6%) cases. There were 152 (63.3%) patients whose LOS was two days or fewer. One hundred twenty-seven (52.9%) patients were shifted to the critical care area (front) of ED. 67 (27.9%) were shifted to non-critical area (stepdown), and only 46 (19.2%) belonged to resuscitation, respectively. One hundred seventy-nine (74.6%) patients recovered and were discharged, but 47 patients (19.6%) died during the study period; only 14 (5.8%) of those patients had left against medical advice without justification. Categories of adult patients were stratified into three groups: 115 (47.9%) had sepsis, 68 (28.3%) had severe sepsis, and 57 (23.8%) had septic shock individually.

Associations with sepsis, severe sepsis, and septic shock are discussed in Table 2. A majority of our patients (n = 143; 59.6%) were older than 50 years. A majority of cases (n = 152; 63.3%) had hospital LOS ≤ 2 days. The ESI was also calibrated, and most patients (n = 154; 64.2%) were in category II. However, 90 patients (78.3%) had symptoms of sepsis (P < .0001). There was a mortality rate of 19.6% (n = 47). However, 30 of 47 (68%) mortality cases were of septic shock (P < .0001).

**Table 2:** Association of remarks with different demographics and study characteristics

	Sepsis	Severe Sepsis	Septic Shock	Total	P-value
	f (%)	f (%)	f (%)	f (%)	
Sex					
Men	71 (61.7%)	39 (57.4%)	29 (50.9%)	139 (57.9%)	.395
Women	44 (38.3%)	29 (42.6%)	28 (49.1%)	101 (42.1%)	
Total	115 (100%)	68 (100%)	57 (100%)	240 (100%)	
Age groups					
≤ 50 Years	61 (53%)	23 (33.8%)	13 (22.8%)	97 (40.4%)	<0.001
> 50 Years	54 (47%)	45 (66.2%)	44 (77.2%)	143 (59.6%)	
Total	115 (100%)	68 (100%)	57 (100%)	240 (100%)	
Length of hospital stay					
≤ 2 days	88 (76.5%)	31 (45.6%)	33 (57.9%)	152 (63.3%)	<0.001
> 2 days	27 (23.5%)	37 (54.4%)	24 (42.1%)	88 (36.7%)	
Total	115 (100%)	68 (100%)	57 (100%)	240 (100%)	
Disposition					
Resuscitation	23 (20%)	12 (17.6%)	11 (19.3%)	46 (19.2%)	<0.001
Front	59 (51.3%)	40 (58.8%)	28 (49.1%)	127 (52.9%)	
Step down	33 (28.7%)	16 (23.5%)	18 (31.6%)	67 (27.9%)	
Total	115 (100%)	68 (100%)	57 (100%)	240 (100%)	
Fate					
Discharged	108 (93.9%)	48 (70.6%)	23 (40.4%)	179 (74.6%)	<0.001
Expired	0 (0%)	17 (25%)	30 (52.6%)	47 (19.6%)	
Left against medical advice	7 (6.1%)	3 (4.4%)	4 (7%)	14 (5.8%)	
Total	115 (100%)	68 (100%)	57 (100%)	240 (100%)	

**Figure 1:**
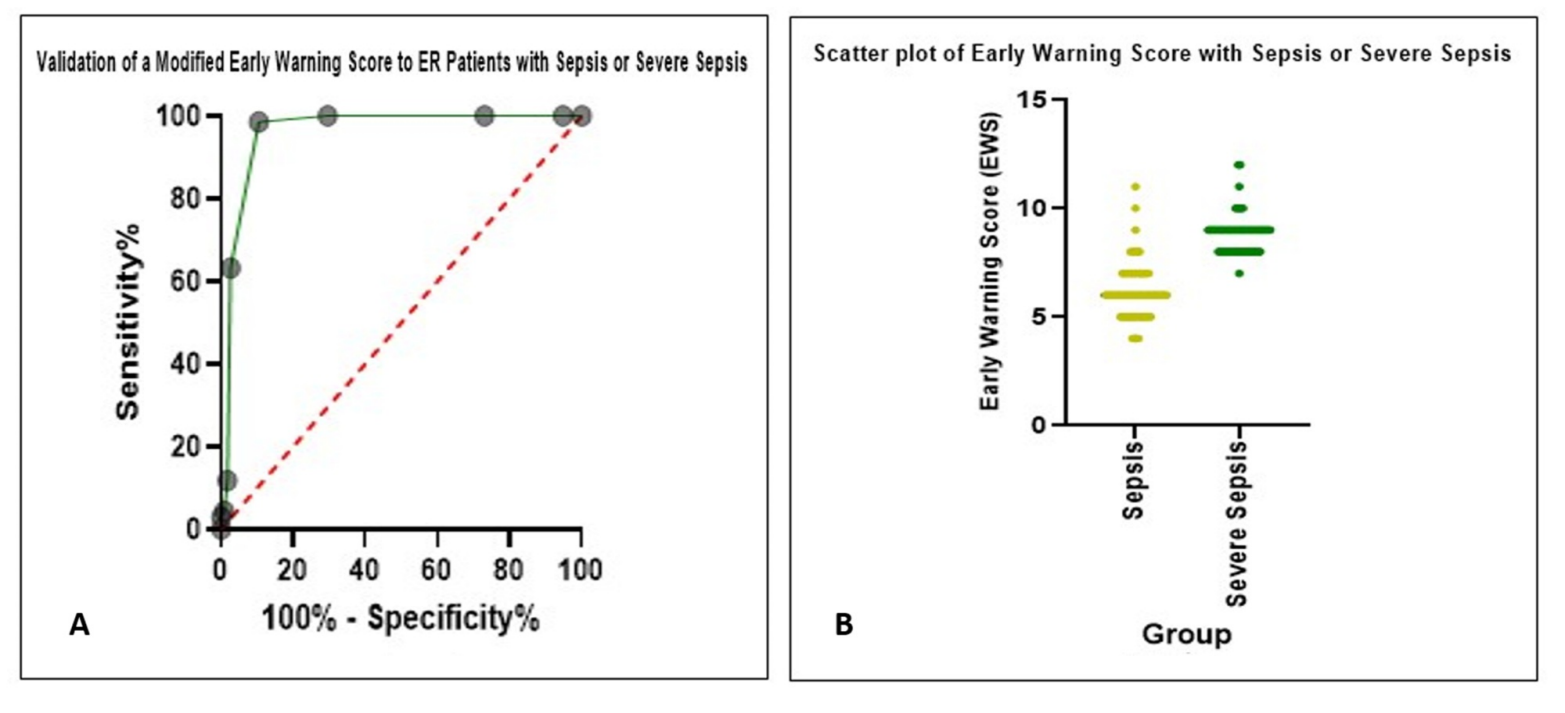
Area under the curve (A) and scatter plot (B) of validation of early warning score at triage with sepsis and severe sepsis

The AUC for EWS to identify the patients with sepsis and severe sepsis at risk is 0.96 (95% CI: 0.933 to 0.989). An EWS >7 is best to detect the cases with sepsis and severe sepsis with a 98.5% Se (95% CI: 92.13 to 99.92) and 89.57% Sp (95% CI: 82.64 to 93.93), with a likelihood ratio of 9.44 (Table 3; Figure 1).

**Table 3:** Sensitivities and specificity of validation of early warning score at triage with sepsis or severe sepsis Abbreviations: EWS = early warning score, CI = confidence interval

EWS cut-off ≥	Sensitivity %	95% CI	Specificity %	95% CI	Likelihood ratio
4	100	94.65% to 100.0%	5.217	2.413% to 10.92%	1.055
5	100	94.65% to 100.0%	26.96	19.69% to 35.71%	1.369
6	100	94.65% to 100.0%	70.43	61.54% to 78.01%	3.382
7	98.53	92.13% to 99.92%	89.57	82.64% to 93.93%	9.442
8	63.24	51.36% to 73.70%	97.39	92.61% to 99.29%	24.24
9	11.76	6.083% to 21.53%	98.26	93.88% to 99.69%	6.765
10	4.412	1.202% to 12.19%	99.13	95.24% to 99.96%	5.074
11	2.941	0.5226% to 10.10%	100	96.77% to 100.0%	

**Figure 2:**
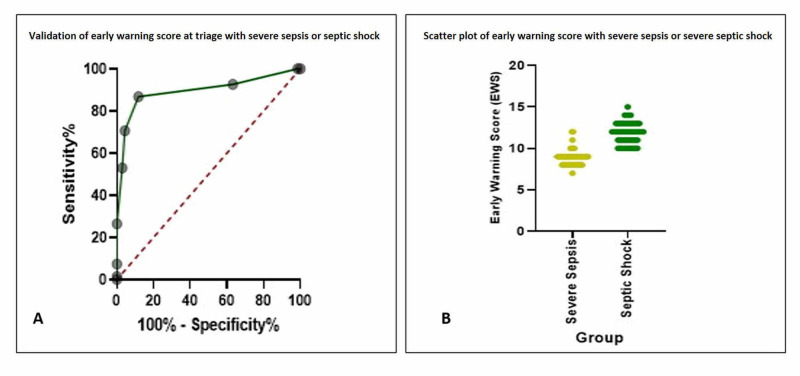
Area under the curve (A) and scatter plot (B) of validation of early warning score at triage with severe sepsis or septic shock Abbreviation: ER, emergency room

While elaborating for severe sepsis and septic shock on EWS, we found the AUC at 0.895 (95% CI: 0.836 to 0.953), and a score of >9 was best to detect cases with an 86.76% Se (95% CI: 76.72 to 92.88) and an 88.24% Sp (95% CI: 78.47 to 93.92), with a likelihood ratio of 7.375 (Table 4; Figure 2).

**Table 4:** Sensitivities and specificity of validation of early warning score at triage with sepsis or septic shock Abbreviations: EWS = early warning score, CI = confidence interval

EWS cut-off ≥	Sensitivity %	95% CI	Specificity %	95% CI	Likelihood ratio
7	100	94.65% to 100.0%	1.471	0.07543% to 7.871%	1.015
8	92.65	83.91% to 96.82%	36.76	26.30% to 48.64%	1.465
9	86.76	76.72% to 92.88%	88.24	78.47% to 93.92%	7.375
10	70.59	58.89% to 80.08%	95.59	87.81% to 98.80%	16
11	52.94	41.24% to 64.33%	97.06	89.90% to 99.48%	18
12	26.47	17.45% to 38.01%	100	94.65% to 100.0%	-
13	7.353	3.181% to 16.09%	100	94.65% to 100.0%	-
14	1.471	0.07543% to 7.871%	100	94.65% to 100.0%	-

## Discussion

Sepsis is a common ED presentation and a leading cause of mortality among severely ill patients [17]. Early identification and initiation of adequate therapy are essential for surviving severe sepsis or septic shock. Therefore, EWS was used for the early diagnosis of sepsis in the emergency department. The objective of our study was to collect information regarding the utilization of EWS in timely identifying the sick patients at triage.

There are multiple scoring criteria for early and rapid identification of septic patients at triage such as sequential organ failure assessment and acute physiology and chronic health evaluation; however, other than physiological factors, most need laboratory parameters, which are almost impossible to obtain from sick patients at busy triage [18]. This score will not only help physicians in early detection and deterioration of septic patients but it will also guide our triage nurse to identify sepsis patient early and can start to initiate and follow their system algorithms that suggest appropriate interventions and management and prevent further deterioration to reduce mortality and LOS [19-21].

With this prospective study, we found that EWS is a sensitive tool in the early identification of sepsis, severe sepsis, and septic shock and may be used to detect a patient at risk. With scores of ≥ 7 with excellent Se of 98.5% and good Sp of 89.57%, these results are better than a similar study from Keep et al. [22]. Similarly, analyzing the same data, using EWS for severe sepsis and septic shock, we found promising results of ≥ 9 having a Se of 86.76% and an Sp of 88.24%. Hence, we may predict that patients with a high EWS are likely to be sicker at triage with more deranged physiological and clinical parameters; however, cut-off values of EWS for sepsis and septic shock are different.

Regarding the age and gender variables, we found similar patterns as Asghar et al. [23] and Morr et al. [24]. Elderly patients aged > 50 years were more prone to sepsis [25-28].

EWS can play a key role in the early diagnosis and management of sepsis because speciﬁc markers for sepsis are not accessible at triage, so a statistically valid, practical, and accurate scoring system could potentially save lives and improve outcomes, morbidity and mortality, for septic patients. Our mean average EWS was 8.2 ± 2.6 (range, 7 to 23), which helps identify all sorts of sepsis early [23,24,26-29].

## Conclusions

An EWS score of >7 at ED presentation has high sensitivity and specificity for sepsis and severe sepsis. In contrast, an EWS >9 has high Se and Sp for severe sepsis and septic shock. Therefore, EWS could be a valuable tool for early identification and timely management of sepsis, severe sepsis, and septic shock.
